# Hammersmith Infant Neurological Examination at 3 Months in Infants at Risk for Congenital Infections: A Cohort Study

**DOI:** 10.1111/jpc.70273

**Published:** 2025-12-31

**Authors:** Karen Cristine Oliveira de Azambuja, Amanda de Arguelho Oliveira Arguelho, Meyene Duque Weber, Lorrainy Marques da Silva Dutra, Tathiana Ghisi de Souza, Daniele Soares‐Marangoni

**Affiliations:** ^1^ Graduate Program in Movement Sciences Federal University of Mato Grosso do Sul Campo Grande Mato Grosso do Sul Brazil; ^2^ Graduate Program in Health and Development Federal University of Mato Grosso do Sul Campo Grande Mato Grosso do Sul Brazil; ^3^ Health Institute Federal University of Mato Grosso do Sul Campo Grande Mato Grosso do Sul Brazil; ^4^ Pediatrics Department Jundiaí Medical School Jundiaí São Paulo Brazil

**Keywords:** child development, infant, neurological examination, prenatal care, risk factors

## Abstract

**Introduction:**

STORCH refers to a group of congenital infections (syphilis, toxoplasmosis, rubella, cytomegalovirus and herpes) that can impact the central nervous system. As clinical signs may not appear until several months or years after birth, the early detection of risk in STORCH‐exposed infants has been challenging, and the use of sensitive tools in this population is understudied.

**Objective:**

To compare STORCH‐exposed infants with non‐exposed controls using the Hammersmith Infant Neurological Examination (HINE) at 3 months of age.

**Methods:**

This is an observational cohort study. A total of 60 infants were included and equally allocated into two groups: an exposed group, whose mothers had a clinically confirmed diagnosis of a classic STORCH infection during pregnancy, and a non‐exposed control group, whose mothers did not present STORCH infections during gestation. At 3 months of age (13.83 ± 1.09 weeks post‐term), infants were assessed using the HINE. Group comparisons were performed for the global score, subscores across the five scorable domains (cranial nerve function, posture, spontaneous movements, tone and reflexes and reactions), number of asymmetries and risk of cerebral palsy.

**Results:**

The exposed group showed lower global scores and lower subscores in most HINE domains compared to controls, along with a higher frequency of asymmetries and an increased proportion of infants classified as at high risk for cerebral palsy.

**Conclusion:**

Infants prenatally exposed to STORCH infections showed an increased risk of impairment based on the HINE when compared to controls. Potential neurological limitations were detectable in the exposed group at 3 months of age.

## 1 Introduction

Congenital infections refer to infections acquired by the fetus or newborn through vertical transmission [1]. The acronym STORCH (or TORCHS) encompasses the classic group of these infections, referring to (S) syphilis, toxoplasmosis (T), others (O), rubella (R), cytomegalovirus (C) and herpes simplex (H) [2]. Among them, congenital syphilis, toxoplasmosis and cytomegalovirus are the most prevalent worldwide, particularly in low‐ and middle‐income countries, and represent a major public health concern [3, 4, 5].

As brain calcifications and severe congenital anomalies, including microcephaly, are among the main outcomes of fetal exposure to these infections, they have been considered major causes of permanent disability in children, including cerebral palsy. However, congenital infections are often asymptomatic at birth, and compromised nervous system integrity may not be detectable in the first months of life [6].

Internationally recommended tools for the early detection of neurodevelopmental impairments before 5 months of age, particularly cerebral palsy, include Prechtl's General Movements Assessment (GMA) [7], the Hammersmith Infant Neurological Examination (HINE) [8] and neonatal magnetic resonance imaging (MRI). The combined use of these tools is considered optimal for accurately identifying infants at risk [9]. However, in many clinical settings, access to MRI and professionals certified in the GMA is limited or absent. In such contexts, the HINE provides a practical, low‐cost and easily trainable alternative for early assessing the risk of cerebral palsy and other neurodevelopmental impairments.

The HINE has been applied in diverse clinical populations, including preterm and/or low‐birth weight infants [10], infants with congenital Zika syndrome [11, 12], infants exposed to SARS‐CoV‐2 [13] and those with bronchopulmonary dysplasia [14]. Despite this relatively broad application, to our knowledge, no study has evaluated neurological responses using the HINE in infants exposed to classical STORCH infections.

To determine whether infants prenatally exposed to STORCH exhibit measurable neurological limitations and risk of cerebral palsy as early as 3 months of corrected age, we compared exposed infants with non‐exposed controls using the HINE. By providing early evidence of potential neurological problems in this population, this study contributes to identifying at‐risk infants and supporting timely intervention in settings where these infections are prevalent.

## 2 Methods

### 2.1 Study Design

This is an observational small cohort study approved by the Human Research Ethics Committee of the Federal University of Mato Grosso do Sul (protocol 67923223.7.0000.0021).

### 2.2 Setting

Participants were recruited from the database laboratory of the Institute of Research, Teaching and Diagnostics (Instituto de Pesquisas, Ensino e Diagnósticos da APAE)—IPED/APAE in Campo Grande, Mato Grosso do Sul state, Brazil. The IPED/APAE laboratory is a state‐level reference centre for maternal STORCH screening tests. The state of Mato Grosso do Sul has 2.757.013 inhabitants [15]. Recruitment also occurred via active search at the Maria Aparecida Pedrossian University Hospital (HUMAP). Data collection was conducted at the research laboratory of the involved University or at the participant's home when necessary. Recruitment and data collection were conducted between January 2023 and November 2024.

### 2.3 Participants and Eligibility Criteria

The study included three‐month‐old infants by convenience sampling, divided into two groups or cohorts: (cohort A) an *exposed group*, whose mothers had a clinically confirmed diagnosis of syphilis, toxoplasmosis or cytomegalovirus infections during pregnancy, and (cohort B) a *non‐exposed group*, whose mothers had no clinical diagnosis of STORCH infections during pregnancy. The clinical information for both groups was retrieved from medical prenatal care records and records from the IPED/APAE database.

Inclusion criteria consisted of 3‐month‐old infants (12–15 weeks post‐term, corrected for prematurity when necessary), of both sexes, with confirmed prenatal exposure to STORCH, from the state of Mato Grosso do Sul, and who were clinically stable. Exclusion criteria consisted of infants who were under sedation or using central nervous system depressant medications, infants who were not within the appropriate age range for assessment and those with inconsistencies in neonatal screening results for STORCH. Infants in both groups were matched according to gestational age classification (full‐term, late to moderate preterm, very preterm, extremely preterm).

### 2.4 Assessment Tool

Infants underwent the HINE assessment at 3 months of age (12–15 weeks post‐term), which scores 26 items across five domains: cranial nerve function, posture, spontaneous movements, tone and reflexes and reactions. Each item is rated on a 0–3 scale, with 3 indicating optimal performance, which yields a maximum global score of 78 points. Additionally, the number of items with asymmetric responses is documented [8].

At 3 months of age, a global HINE score ≥ 67, ≥ 62 and ≥ 58 is considered optimal for, respectively, term‐born, late preterm and very preterm and extremely preterm infants. Global scores below this threshold are classified as suboptimal and may indicate an increased risk for motor impairment. A global score ≤ 56 at this age is highly predictive of cerebral palsy [16]. The presence of up to three asymmetries is considered typical, whereas four or more suggest risk of hemiplegic cerebral palsy or asymmetric hand function [16, 17, 18].

The HINE assessments were conducted by two experienced paediatric physical therapists, each having completed a total of 24 h of combined theoretical and practical HINE training courses. Assessments lasted approximately 15 min and were scheduled to avoid vaccination days, the infant's nap and feeding times. During the assessment, infants wore only diapers or light clothing that allowed full visibility and unrestricted movement of the upper and lower limbs.

### 2.5 Statistical Analysis

Data was analysed using SPSS 23.0. Assumptions of normality and homogeneity of variances were assessed using the Kolmogorov–Smirnov and Levene's tests, respectively. Descriptive statistics were calculated to characterise the sample, including means, standard deviations and minimum and maximum values for continuous variables, as well as absolute frequencies and proportions for categorical variables. Between‐group differences in sample characteristics were examined using independent‐samples t‐tests.

Group comparisons for the HINE assessment (global score, subscores of the 5 domains and number of asymmetries) were performed using the Mann–Whitney test. A chi‐square test was applied to compare the proportion of infants at high risk for cerebral palsy between the groups.

Effect sizes were calculated and reported for all analyses to complement statistical significance testing. Effect sizes were calculated for all analyses: Cohen's *d* for continuous variables (small 0.20–0.49, medium 0.50–0.79, large ≥ 0.80), *r* for Mann–Whitney tests (small 0.10–0.29, medium 0.30–0.49, large ≥ 0.50) and Cramér's *V* for *χ* ^2^ tests, using the same thresholds as *r*. All nonparametric effect size calculations were performed in Python (version 3.12.3) using its standard mathematical library. All statistical tests adopted a two‐sided significance level of 5% (*α* = 0.05).

## 3 Results

**FIGURE 1:**
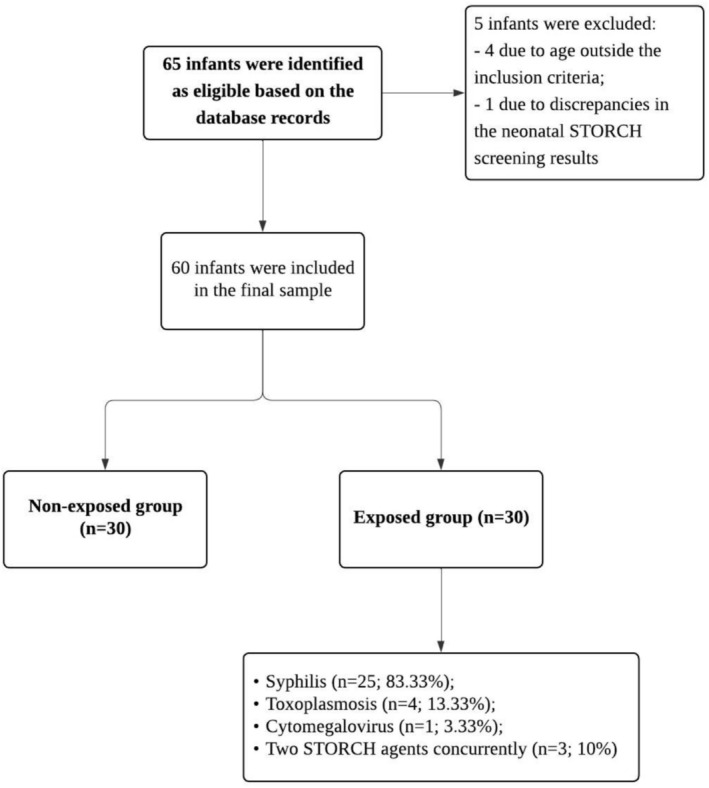
Flowchart showing the selection of participants.

Of the 65 eligible infants, 60 were included in the final sample, with 30 in each group (exposed and non‐exposed) (Figure 1).

### 3.1 Sample Characterisation

Among the included infants, 53.33% (*n* = 32) were male. Syphilis was the most frequent infection (Figure 1). Eighty percent (*n* = 48) were born at term. The proportion of preterm infants (20%) was identical in both groups (*n* = 6 each), consistent with the matching based on gestational age classification. Their mean gestational age was 32.67 ± 3.67 and 34.33 ± 1.63 in the exposed and non‐exposed group, respectively. None of the infants underwent brain imaging exams. Table 1 presents further details of the sample characteristics. The groups showed similar characteristics, except for head circumference at birth, which was slightly smaller in the STORCH‐exposed group compared to the non‐exposed group.

**TABLE 1:** Characteristics of the sample.

Characteristics	Groups *M* ± SD		Total (*n* = 60)	*p*	Cohen's *d*
	Exposed to STORCH (*n* = 30)	Non‐exposed (*n* = 30)			
Gestational age (weeks)	37.65 ± 3.37	37.59 ± 2.17	38.80 ± 2.69	0.85	0.00
Infant age at assessment (weeks post‐term)	13.96 ± 1.31	13.67 ± 0.87	13.83 ± 1.09	0.16	0.26
Birth weight (g)	2953.97 ± 777.26	3030.45 ± 540.07	2991.56 ± 666.61	0.66	0.11
*z*‐score for head circumference at birth	0.35 ± 1.20	1.01 ± 0.79	0.73 ± 0.14	0.01	0.64
*z*‐score for head circumference at the assessment	0.39 ± 1.72	0.94 ± 0.93	0.73 ± 1.82	0.13	0.39
Apgar score at 1 min	8.58 ± 0.70	8.11 ± 1.37	8.24 ± 1.32	0.07	0.43
Apgar score at 5 min	9.31 ± 0.62	9.19 ± 0.83	9.22 ± 0.74	0.42	0.62
Mother's age (years)	26.53 ± 6.47	29.93 ± 6.19	28.15 ± 6.52	0.06	0.53
Per capita income (US$)	167.65 ± 145.83	218.20 ± 265.30	192.93 ± 142.38	0.10	0.23

*Note*: Head circumference *z* scores based on the INTERGROWTH‐21st (Villar et al. 2015) [19]; mother's age presented in years; per capita income was obtained in Brazilian Real (R$) and subsequently converted into United States dollars (US$) using the exchange rate in effect on August 20, 2025. *p* value using independent *t* tests.

Abbreviations: *M*, mean; SD, standard deviation.

### 3.2 Group Comparisons on the HINE

Significant differences between the groups were found for the HINE global score (*U* = 198.00) and for the subscores related to cranial nerve function (*U* = 257.50), movements (*U* = 315.00), tone (*U* = 245.50) and reflexes and reactions (*U* = 296.50), with the exposed group showing lower scores than the non‐exposed group. No significant difference was found in the posture subscore between groups (*U* = 346.00) (Table 2).

**TABLE 2:** Differences in HINE scores at 3 months of age between the STORCH‐exposed and non‐exposed groups (*n* = 60).

HINE	Groups median (min–max)		*p*	*r*
	Exposed to STORCH (*n* = 30)	Non‐exposed (*n* = 30)		
Global scores	59.0 (50.0–69.0)	65.2 (51.2–72.0)	< 0.001	−0.48
Cranial nerve function	13.0 (10.0–15.0)	14.0 (11.0–15.0)	< 0.001	−0.36
Posture	12.0 (8.0–17.0)	13.2 (7.5–18.0)	0.12	−0.19
Movements	6.0 (2.0–6.0)	6.0 (6.0–6.0)	< 0.001	−0.25
Tone	21.7 (19.0–24.0)	23.5 (18.0–24.0)	< 0.001	−0.39
Reflexes and reactions	8.0 (3.0–11.0)	9.0 (4.0–12.0)	0.02	−0.29
Number of asymmetries	2.0 (0–4.0)	1.0 (0–2.0)	< 0.001	−0.44

Abbreviations: max, maximum value; min, minimum value; *p*, independent sample comparison using the Mann–Whitney test; *r*, effect size.

A higher proportion of infants in the exposed group presented suboptimal global scores (63.6%; *n* = 29) compared to the non‐exposed group (36.4%; *n* = 16) (*χ* ^2^(1) = 12.27; *p* = 0.001; Cramer's *V* = 0.45).

Between‐group differences were also observed in the number of asymmetries (*U* = 217.50), which was higher in the exposed group compared to the non‐exposed group (Table 2). More than three asymmetries were observed in one infant (1.7%), who was in the exposed group.

**FIGURE 2:**
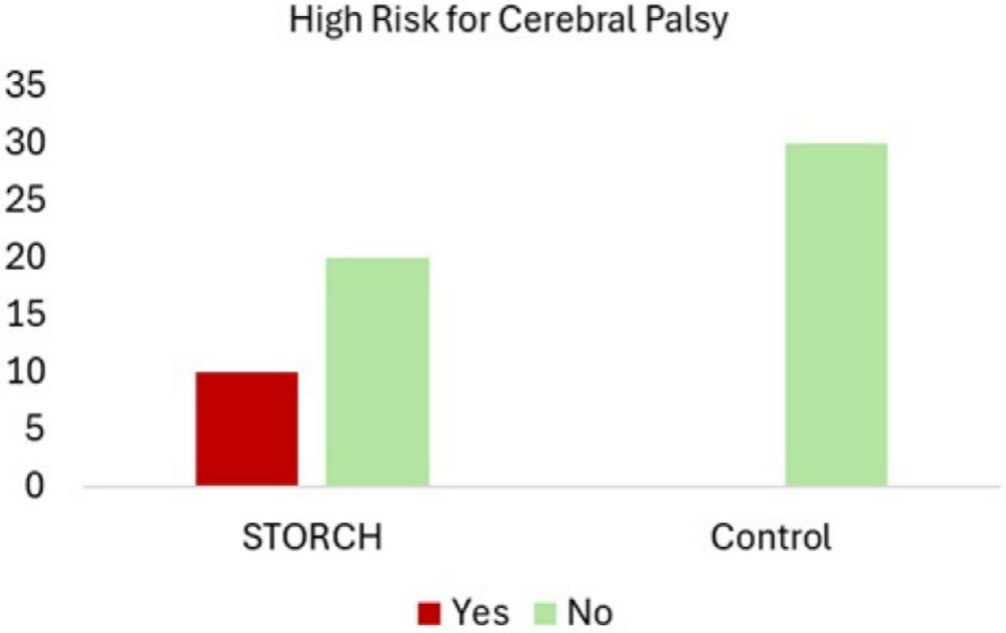
Number of infants with and without high risk for cerebral palsy according to group.

There was also a higher proportion of infants at high risk for cerebral palsy in the STORCH‐exposed group compared to controls (*χ* ^2^(1) = 12.000; *p* = 0.01; *V* = 0.63). All infants identified as high risk (16.7%; *n* = 10) belonged to the exposed group (Figure 2).

## 4 Discussion

The present study verified differences in aspects of the neurological function assessed with the HINE between STORCH‐exposed and non‐exposed controls at 3 months of age. Exposed infants showed lower HINE scores, a greater number of asymmetries and a higher risk of cerebral palsy compared with controls. Effect sizes were large for the higher proportion of exposed infants with suboptimal HINE scores and for the proportion classified as at high risk for cerebral palsy. Syphilis was the most frequent infection, followed by toxoplasmosis and cytomegalovirus; infants exposed to rubella and herpes were not identified. These incidences align with data provided in the literature [3, 5, 20, 21]. To our knowledge, this is the first study to report results of the HINE in a population exposed to classic STORCH infections, specifically syphilis, toxoplasmosis and cytomegalovirus.

Previous studies have examined HINE outcomes in infants prenatally exposed to Zika virus, a well‐known congenital infection with significant clinical and epidemiological relevance. In a study on the accuracy of the HINE in Zika‐exposed infants aged 3 to 24 months, the exposed group scored significantly lower in the ankle dorsiflexion test and showed a trend toward lower global scores compared with the non‐exposed controls [12]. A study following children with Zika virus‐associated microcephaly beyond 2 years identified severe neurological impairments on the HINE, affecting both movement and posture [11]. In our study, although head circumference did not differ between groups at the time of assessment, STORCH‐exposed infants scored lower across all HINE items except posture. This finding may suggest that HINE can identify early neurological deficits in STORCH‐exposed infants even when structural abnormalities, such as microcephaly, are absent or not evident. By comparing our results with findings in Zika‐exposed populations, we highlight the broader applicability of the HINE as a potential tool for detecting early neuromotor dysfunction in infants affected by various congenital infections.

It is important to note that infants can demonstrate compensatory head growth between birth and three months of age [22, 23]. Hence, the absence of group differences in head circumference at 3 months suggests that the lower head circumference in the exposed infants at birth may not have been a significant clinical problem. Additionally, the absence of group differences in posture may reflect the inherent difficulty of this domain, even among lower‐risk infants. The posture domain assesses, for instance, head control, which typically remains incomplete at 3 months, and trunk control, which is still immature in normally developing infants at this age [24]. This may indicate the importance of age‐appropriate interpretation of the individual HINE domains, particularly as early as 3 months of age. Moreover, this observation reinforces the need for repeated assessments over the first year of life to capture emerging motor skills and to distinguish between transient immaturity and early indicators of neuromotor impairment.

We observed significant group differences in the number of asymmetries, with STORCH‐exposed infants presenting a higher count than non‐exposed infants. One infant from the exposed group exhibited four asymmetries, a pattern associated with an increased risk of hemiplegia [17] and asymmetric hand function [18], while the maximum number of asymmetries observed in the non‐exposed group was three. HINE asymmetry scores have been shown to provide valuable information for distinguishing mild forms of cerebral palsy from typical development [16, 17], even when global scores are not low [16, 18]. Our findings indicate that the STORCH‐exposed infants had neuromotor profiles closer to the threshold for impairment, which suggests early vulnerability even in the absence of overt structural abnormalities compared to their peers. This reinforces the relevance of including asymmetry assessment in early neurological evaluations and supports the use of the HINE for identifying infants who may benefit from targeted early interventions.

To date, to the best of our knowledge, the only study that has assessed aspects of neurological function in infants exposed to STORCH infections using tools recommended by international guidelines [9] was an exploratory report, conducted without a control group, which employed the GMA. In that study, 15 infants aged 3–4 months who had been exposed to syphilis or toxoplasmosis displayed a reduced motor repertoire and abnormal spontaneous movements, indicating an increased risk for neurodevelopmental problems [25]. Our findings complement this prior report by providing controlled evidence that STORCH‐exposed infants may exhibit measurable neurological deficits and risk of cerebral palsy as early as 3 months of age. By applying the HINE, a standardised and widely accessible assessment tool, we were able to quantify specific domains of neuromotor function and detect early deviations that might signal increased risk for later developmental impairments, particularly cerebral palsy. This study, therefore, strengthens the rationale for early monitoring and potential early intervention strategies in this vulnerable population.

The literature indicates that infants exposed to STORCH infections are at increased risk for developmental impairment that includes not only cerebral palsy but also autism spectrum disorder and global developmental delays, among others [26, 27]. These adverse outcomes often remain undetectable in early infancy and may only become apparent years later [6, 26, 27]. Hence, early identification of neurological dysfunction, as proposed in the present study, represents a feasible strategy to provide timely intervention for STORCH‐exposed infants. The HINE, as a low‐cost and easily implemented tool, allows healthcare professionals in diverse clinical settings to detect early risk of neuromotor impairment. This approach is particularly relevant in low‐ and middle‐income countries, where access to advanced medical technologies and trained professionals for other sensitive assessment tools, such as the GMA, is still restricted.

A limitation in this study is the relatively small sample size. This restricts the external validity and generalizability of the findings, as it may not capture the full heterogeneity of infants exposed to STORCH infections. Second, the absence of longitudinal follow‐up limits the interpretation of the results, since some neurological findings at 3 months of age may represent transient immaturity rather than permanent dysfunction. Longitudinal monitoring would be essential to determine whether the observed differences persist and whether they translate into clinically significant developmental outcomes. Third, due to constraints in available human resources, blinding of assessors to the infants' clinical history was not feasible, which may have increased the risk of observer bias during the examinations. Finally, although the HINE is a validated and widely accessible tool, combining it with complementary assessments, such as the GMA and/or neuroimaging, could provide a more comprehensive understanding of early neurological function in this population. Future large‐scale cohort studies with longer follow‐up periods and multimodal approaches will be necessary to establish the predictive value of the HINE in infants exposed to STORCH infections.

## 5 Conclusion

In conclusion, this study demonstrates that infants prenatally exposed to syphilis, toxoplasmosis and cytomegalovirus infections may exhibit measurable neurological limitations as early as 3 months of age, as assessed by the HINE. They demonstrated a greater risk of impairment, particularly cerebral palsy, relative to controls, even in the absence of evident structural abnormalities, such as microcephaly. These findings reinforce the value of the HINE as a low‐cost, practical and sensitive tool for early detection of neuromotor impairment in biologically vulnerable populations, particularly in low‐ and middle‐income settings.

## Funding

This work was supported by the Coordination for the Improvement of Higher Education Personnel—CAPES, Brazil [001], Federal University of Mato Grosso do Sul, Brazil [001] and National Council for Scientific and Technological Development—CNPq [404346/2023‐5].

## Conflicts of Interest

The authors declare no conflicts of interest.

## Data Availability

The data that support the findings of this study are available on request from the corresponding author. The data are not publicly available due to privacy or ethical restrictions.
